# Conjoint analysis of circulating tumor cells and solid tumors for exploring potential prognostic markers and constructing a robust novel predictive signature for breast cancer

**DOI:** 10.1186/s12935-021-02415-8

**Published:** 2021-12-25

**Authors:** Xuan Li, Hefen Sun, Qiqi Liu, Yang Liu, Yifeng Hou, Wei Jin

**Affiliations:** 1 Department of Breast Surgery, Key Laboratory of Breast Cancer in Shanghai, Fudan University Shanghai Cancer Center, Shanghai, 200032 China; 2 Department of Oncology, Shanghai Medical College, Fudan University, Shanghai, 200032 China

**Keywords:** Circulating tumor cells, Breast cancer, Molecular classification, Immune signature, Prognostic factor

## Abstract

**Background:**

Distance metastasis is the leading cause of death for breast cancer patients, and circulating tumor cells (CTCs) play a key role in cancer metastasis. There have been few studies on CTCs at the molecular level due to their rarity, and the heterogeneity of CTCs may provide special information for solid tumor analysis.

**Methods:**

In this study, we used the gene expression and clinical information of single-cell RNA-seq data of CTCs of breast cancer and discovered a cluster of epithelial cells that had more aggressive characteristics. The differentially expressed genes (DEGs) between the identified epithelial cells cluster and others from single-CTCs were selected for further analysis in bulk sequence data of solid breast cancers.

**Results:**

Eighteen genes closely related to the specific CTC epithelial phenotype and breast cancer patient prognosis were identified. Among these 18 genes, we selected the GARS gene, which has not been studied in breast cancer, for functional research and confirmed that it may be a potential oncogene in breast cancer. A risk score was established by the 18 genes, and a high-risk score was strongly associated with a high metastasis rate and poor survival prognosis in breast cancer. The high-risk score group was related to a defective immune infiltration environment in breast cancer, and the immune checkpoint therapy response rate was lower in this group. The drug-sensitive analysis shows that the high-risk score patients may be more sensitive to AKT-mTOR and the cyclin-dependent kinase (CDK) pathways drugs than low-risk score patients.

**Conclusions:**

Our 18-gene risk score shows good prognostic and predictive values and might be a personalized prognostic marker or therapy guide marker in breast cancer patients.

**Supplementary Information:**

The online version contains supplementary material available at 10.1186/s12935-021-02415-8.

## Introduction

Breast cancer has the highest incidence of tumors among females in the US [1]. Although breast cancer accounts for the second highest mortality due to cancer in women, breast cancer patients in the early-stage still have a better long-term survival rate. Distance metastasis is the leading cause of death for breast cancer patients, and the American Joint Committee on Cancer (AJCC) shows that stage IV patients only had a 5-year survival rate of less than 30% [2]. In the conventional model, the unlimited proliferation of cancer cells disseminates and adapts to distant sites to contribute to metastasis [3–6]. Epithelial cancer cells undergo epithelial-to-mesenchymal transition (EMT) to peripheral vessels, and then these cells are renamed circulating tumor cells (CTCs). The survival and mesenchymal-to-epithelial transition (MET) at distant sites of CTCs are considered to be involved in metastasis [7]. CTCs are not only considered important “seeds” for distant metastasis but also use self-seeding methods to accelerate tumor growth and angiogenesis processes [8]. An effective capture method and comprehensive analysis of CTCs can help us better study tumors. Currently, thanks to the development of isolation and sequencing technology, CTCs can be better captured, enriched, and detected for clinical use [9, 10].

In recent years, with the development of in-depth sequencing technology in genomics, the drop in price and easy access and exploration of data from large datasets, a large amount of patient gene transcript expression data has been made available to researchers [11]. Many researchers have attempted to find new potential biomarkers or gene sets to better predict the prognosis of patients or classify patients into significant feature groups. Breast cancer is highly heterogeneous and can be identified by the expression states of estrogen receptor (ER), progesterone receptor (PR), and human epidermal growth factor receptor 2 (HER2) into four intrinsic subtypes. Researchers analyzed a large amount of genomic data to reclassify breast cancer patients in a more personalized way, such as the PAM50 classification, which reclassified patients into luminal A, luminal B, HER2-enriched, basal-like and normal like groups [12]. Pommier et al. used gene methylation and gene expression analyses to describe a group of claudin-low tumors from triple-negative breast cancers (TNBCs) comprehensively, which can partially reveal the malignant features of TNBCs [13]. Shao and his team applied genomic analysis in 465 TNBC patients and further classified TNBCs into more suitable categories, which could guide treatment choice and indeed show good guide treatment effects in subsequent clinical trials [14, 15]. Thus, the continuous search for new tumor subtypes is essential to address the heterogeneity of tumors.

Single-cell RNA-sequencing (scRNA-seq) technology can provide deep insight into transcriptomic information at the single-cell level and help reveal unidentified subgroups [16, 17]. In this study, we used scRNA-seq technology in single CTCs to identify a unique subtype that may be closely related to metastasis. Combination analysis of CTCs and bulk primary tumor data help to build a gene set that can better represent a unique subtype in breast cancer. Comprehensive bioinformatics analyses were applied to explore the characteristics of the gene set classification and its potential precision targeted therapy.

## Materials and methods

### RNA-seq and clinical data collection

The training data for breast cancer CTCs were from the Gene Expression Omnibus (GEO) set GSE109761, and 116 single CTCs were enrolled for analysis (https://www.ncbi.nlm.nih.gov/gds/). The criteria for selecting the 116 single CTCs were as follow: (1) Homo sapiens, (2) number of cells: 1, (3) sample type: CTC single. The verification set was GSE144494, and 134 single-cell RNA-sequencing datasets were used. Bulk RNA-seq data and matched clinical information for breast cancer were downloaded from The Cancer Genome Atlas (TCGA) data portal (https://tcga-data.nci.nih.gov/tcga/), METABRIC cohort (https://www.cbioportal.org/) and GEO datasets. Gene mutation data and copy number alteration (CNA) of breast cancer from TCGA were downloaded from UCSC Xena (http://xena.ucsc.edu/).

### Identification and establishment of the risk score

CTC classification was performed using the “Seurat” package in R software, and the criteria for filtering low-quality cells included < 50 genes/cell, < 3 cells/gene and > 5% mitochondrial genes. The package “SingleR” was used to annotate each CTC cluster.

The selection of differentially expressed genes (DEGs) was performed using the “limma" package in R software with a p value less than 0.05 and log2 |fold change| > 1. DEGs selected from CTCs were then subjected to univariate Cox regression analysis in bulk RNA-Seq data of breast cancer from TCGA and GEO. Subsequently, least absolute shrinkage and selection operator (LASSO) regression was performed in R software by using the ‘glmnet’ package to select robust prognostic markers from the results of previous univariate Cox regression analysis. The linear combination of gene expression weighted by regression coefficients (Co-effs) results from multivariate Cox analysis was used to calculate the risk score of patients. The best cutoff of the risk score was dependent on the Youden index of each receiver operating characteristic (ROC) curve. Kaplan–Meier curves and log-rank tests were generated to illustrate the relationship between the survival and risk score groups by SPSS.

### Cell culture and breast cancer specimens

MDA-MB-231, MDA-MB-436, MDA-MB-453, MDA-MB-468, HS-578T, BT-549 and BT-474 cells were purchased from American Type Culture Collection (ATCC). SK-BR-3 and T-47D and the normal mammary epithelial cell line MCF-10A were purchased from the Cell Bank of Type Culture Collection of the Chinese Academy of Science (Shanghai, China). The high lung metastasis potential cell line MDA-MB-231 HM developed from its parental cell line MDA-MB-231 via four cycles of tail vein injections in our laboratory (patent number: 200910174455.4). The high lung metastasis potential cell line MDA-MB-231 LM2 was kindly provided by Dr. Toshiyuki Yoneda (The University of Texas, Houston, US).

All cells were grown in the appropriate medium and cultured at 37 °C in a humidified atmosphere with 5% CO_2_. Total RNA was extracted from the cell lines by using Trizol reagent (Invitrogen).

Sixteen pairs of breast carcinomas and paraneoplastic tissues were randomly collected from patients who underwent surgical treatment for breast cancer at the Fudan University Shanghai Cancer Center. RNA from these tissue samples was extracted by using the AllPrep DNA/RNA/Protein Mini Kit (QIAGEN; Cat. No. 80004) for subsequent PCR analysis. The use of all clinical samples was approved by the Ethics Committee of the Cancer Center of Fudan University.

### Quantitative real-time polymerase chain reaction

The total RNA of cell lines and tissues was immediately reverse transcribed to cDNA by using the PrimeScript RT Reagent Kit (Perfect Real-Time; TaKaRa Biotechnology). The subsequent real-time polymerase chain reaction (RT-PCR) was performed by SYBR Premix Ex Taq (TaKaRa Bio) using an ABI Prism 7900 instrument (Applied Biosystems).

The following sequences were used for our study:

- GARS: F 5′-ATGGAGGTGTTAGTGGTCTGT-3′,
- GARS: R 5′-CTGTTCCTCTTGGATAAAGTGCT-3′,
- GGCX: F 5′-GATGCAAACCACTACTGGTCTG-3′,
- GGCX: R 5′-CCGCAATGAAGTACACAATGAAG-3′,
- RNF139: F 5′-TAGGCTTAATCACAGAGCTACCA-3′,
- RNF139: R 5′-CTGCCAGGACAAACACTGTAT-3′,
- TARS: F 5′-ATTGCCTGTGGAATTAGTCAAGG-3′,
- TARS: R 5′-CACCCATTATGTGAGCACTAGAG-3′,
- GAPDH: F 5′-GGAGCGAGATCCCTCCAAAAT-3′,
- GAPDH: R 5′-GGCTGTTGTCATACTTCTCATGG-3′.

### RNA interference

HS-578T and MDA- MB-231 LM2 cells were transfected with GARS small interfering RNA (siRNA) using Lipofectamine RNAiMAX (ThermoFisher, NO. 13778-150) following the manufacturer’s instructions. All experiments were performed 48 h after transfection. The siRNA sequence for GARS used in this experiment was (GATGGAGTATCTTGCCATT).

### Western blotting

Total cell protein was extracted with RIPA lysis buffer (Thermo Scientific, NO. 78510) with 1% protease inhibitors and phosphatase inhibitors. A total of 20 μg protein was separated using a 10% SDS-PAGE gel and electrotransferred onto PVDF membranes (Millipore Immobilon-P). Membranes were blocked with 10% nonfat milk (Sangon Biotech, NO. A600669-0250) and then incubated with the primary antibody overnight. Following washing of the membranes three times with 0.1% Tween‐20-PBS, membranes were then incubated with anti-mouse anti-rabbit IgG and HRP-linked antibody (Cell Signaling Technology, NO. 7076, NO. 7074) for 2 h at room temperature and visualized with an ECL detection system (Share-bioBiotechnology, NO. SB-WB011). The primary antibodies used in our study are listed in Additional file 1: Table S1.

### Cell proliferation assay

Cell proliferation ability was evaluated by the CCK8 assay (Vazyme, NO. A311-02). Briefly, 100 μl of cell suspension (1.5 × 10^3^ cells per well) was seeded in 96-well plates and cultured at 37 °C and 5% CO_2_ for several days. At the same time of each day, the medium was removed, and CCK8 solution was added to each well and then incubated for 2 h at 37 °C. The absorbance at OD 450 nm of each well was measured with a Bio-rad microplate reader.

### Colony formation assay

The cells were seeded in 6-well plates in culture medium at a density of 1 × 10^3^ per well for several days. Then, the cells were fixed with methanol containing 1% crystal violet for 30 min. The colonies were counted, and the data are presented as the mean ± SD.

### Transwell invasion assay

Cell migration assays were performed in 8 um pore size cell culture insets with transparent PET membranes in 24-well plates (FALCON, NO, 353097). The bottom layer of the inset contained 600 µL culture median with 20% serum, and the upper layer of the inset contained 200 µL serum-free culture median with 5 * 10^4^ cells. After culturing for 5 h for HS-578T cells and 16 h for MDA-MB-231 LM2 cells, the insets were fixed in methanol with crystal violet for half an hour. The procedure for the cell invasion assay was similar to the migration assay, but Matrigel was present outside the inset (CORNING, NO. 35448), and the cell number in the inset was 1 * 10^5^/per well. Migrating or invading cells were detected by counting the crystal violet-stained cells.

### Flow cytometry

Cells were seeded in 6-well plates in culture medium at a density of 1 × 10^6^ per well overnight. Then, the culture medium was removed, and deprived serum medium was used to starve the cells for 24 h to synchronize the cell cycle. After 24 h of serum starvation culture, the cells were replaced with normal medium and cultured for 5 h for HS-578T or 8 h for MDA-MB-231 LM2 cells before being harvested for cell cycle analysis. For cell cycle analysis, cells were harvested and fixed in ice-cold 70% ethanol overnight, stained with propidium iodide (Sangon Biotech, NO. E607306-0200) according to the protocol, and analyzed via flow cytometry (BD Biosciences, USA). The cell cycle G1, S and G2 phases of cells were analyzed by appropriate gating on the distribution plot and analyzed by FlowJo (V10.7.1).

### Biofunction enrichment analysis

The bioinformation of DEGs was identified by the Kyoto Encyclopedia of Genes and Genomes (KEGG) pathway analyses on the DAVID website (https://david.ncifcrf.gov/). Gene set enrichment analysis (GSEA) was performed to explore the potential pathways between the high- and low-risk score groups. Hallmark gene sets and Oncogenic signatures set in GSEA were used and the main parameters used were as follows: Enrichment statistic: weighted; Metric for ranking genes: Signal2Noise; Max size of genes: 500; Min size of genes: 30; and the p value for a false discovery rate (FDR) < 0.05 was considered significantly statistically enriched. The visualization of tumor mutation data was performed using the “maftools” package in R software. GISTIC analysis was applied to assess the copy number variation (CNA) in each group, and a GISTIC value greater than 1 was defined as amplification, while a value less than -1 was defined as deletion [18]. The correlation of genes was analyzed in GeneMANIA (http://genemania.org/), and the top 30 most corelated genes with targeted genes were visualized.

### Correlation of the risk score with the tumor immune environment

The 22 tumor-infiltrated immune cells were calculated by the CIBERSORT algorithm, and the value indicates the fraction of each immune cell type in tumor tissues. Tumor stromal score, immune score, ESTIMATE score, and tumor purity were calculated by the R package “estimate”, and single-sample gene set enrichment analysis (ssGSEA) was used on the scRNA-Seq of CTCs and the different bulk RNA-Seq tumor datasets.

### Drug and risk score interaction analysis

The drug_sensitive_AUC data and the RNA-Seq data of breast cancer cell lines were obtained from the Cancer Therapeutics Response Portal (CTRP) database (http://portals.broadinstitute.org/ctrp/). The relationship of risk score and drug_sensitive_AUC was analyzed by Pearson correlation coefficient in SPSS.

### Statistical analyses

In this study, R software (version 3.6.1), SPSS (version 25) and, Prism 8 were the primary software types used. A two-tailed p value less than 0.05 was used to judge statistic in all our analyses.

## Results

### Special epithelial cell cluster identified in single CTCs

**Fig. 1:**
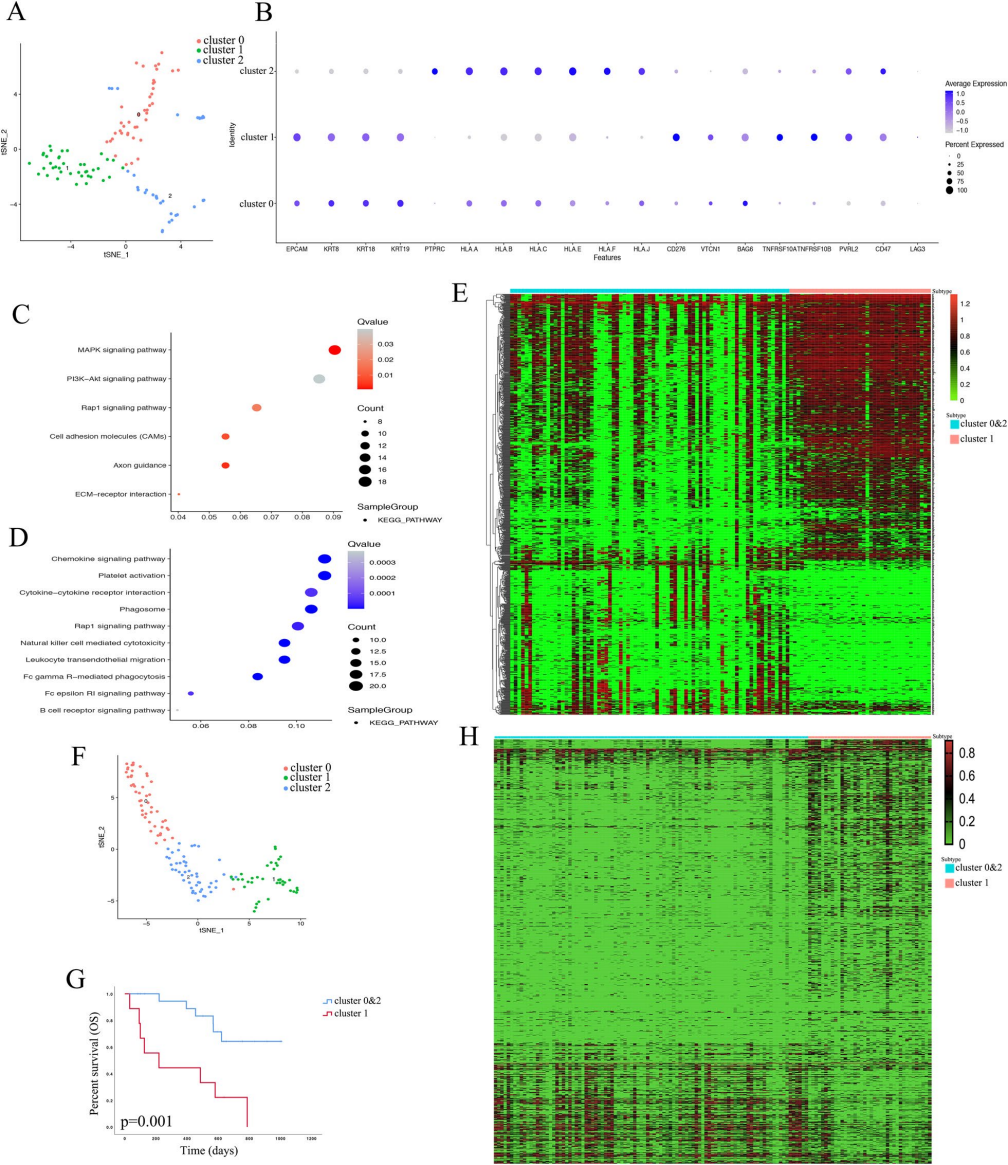
Identify a special cluster with distinct gene expression and outcome in single-CTC cohorts of breast cancer. **A** Analysis of a breast cancer single-CTC RNA-Seq dataset identifies 3 clusters from unsupervised t-SNE clustering in GSE109761. **B** The marker genes expressed in 3 clusters of GSE109761. **C** KEGG pathway analysis of Cluster 1 highly expressed marker genes in GSE109761. **D** KEGG pathway analysis of marker genes with low Cluster 1 expression in GSE109761. **E** The heatmap shows the differentially expressed genes between Cluster 1 and other samples of GSE109761. **F** Analysis of a breast cancer scRNA-Seq dataset identifies 3 clusters from unsupervised t-SNE clustering in GSE144494. **G** Kaplan–Meier (K-M) curve shows the survival difference of Cluster 1 and others of GSE144494. **H** Different expression gene orders of GSE109761 were applied in GSE144494 as a validation set

We applied principal component analysis (PCA) to all genes of the 116 single-cell RNA-sequence datasets of CTCs from GSE109761 by using t-SNE and finally partitioned the samples into three main clusters. The SingleR package annotates the three clusters: epithelial cells (Cluster 0), epithelial cells (Cluster 1), and monocytes (Cluster 2) (Fig. 1A). Analysis of epithelial cell marker genes and immune checkpoint related genes in these three clusters showed that Cluster 1 was different from the other two clusters, as it expressed significant circulating tumor cell markers, low MHC-I related genes, and some high immune checkpoints (Fig. 1B). The highly expressed marker genes in Cluster 1 were enriched in the MAPK, PI3K-AKT, and Rap1 signaling pathways as well as in the cell accession and ECM receptor interaction pathways (Fig. 1C). The low expression marker genes of Cluster 1 were enriched in some immune related pathways, such as the chemokine signaling pathway, cytokine-cytokine receptor interaction, natural killer cell mediated cytotoxicity, F c gamma R-mediated phagocytosis, and B cell receptor signaling pathway (Fig. 1D). The heatmap shows the expression of different marker genes between Cluster 1 and others (Fig. 1E). An independent set includes 135 single-cell RNA-sequence data of CTCs that were collected from GSE144494 to assess subtype reproducibility. PCA and t-SNE also classified the 135 samples into three clusters, and the SingleR package annotated the three clusters to erythrocytes (Cluster 0), epithelial cells (Cluster 1), and erythrocytes (Cluster 2) (Fig. 1F). Patients who had Cluster 1 CTCs had lower overall survival than other patients (Fig. 1G). Applying a gene set (marker genes of Cluster 1 in GSE109761 and the gene order just the same as Fig. 1E) in Cluster 1 and other samples of GSE144494 can clearly reproduce the similar gene expression trend as shown in Fig. 1E (Fig. 1H). In this part, we found a class of CTCs with a significant epithelial cell phenotype, lower immune function, and poor survival prognosis.

### The selection of 18 prognosis-related genes and their verification

**Fig. 2:**
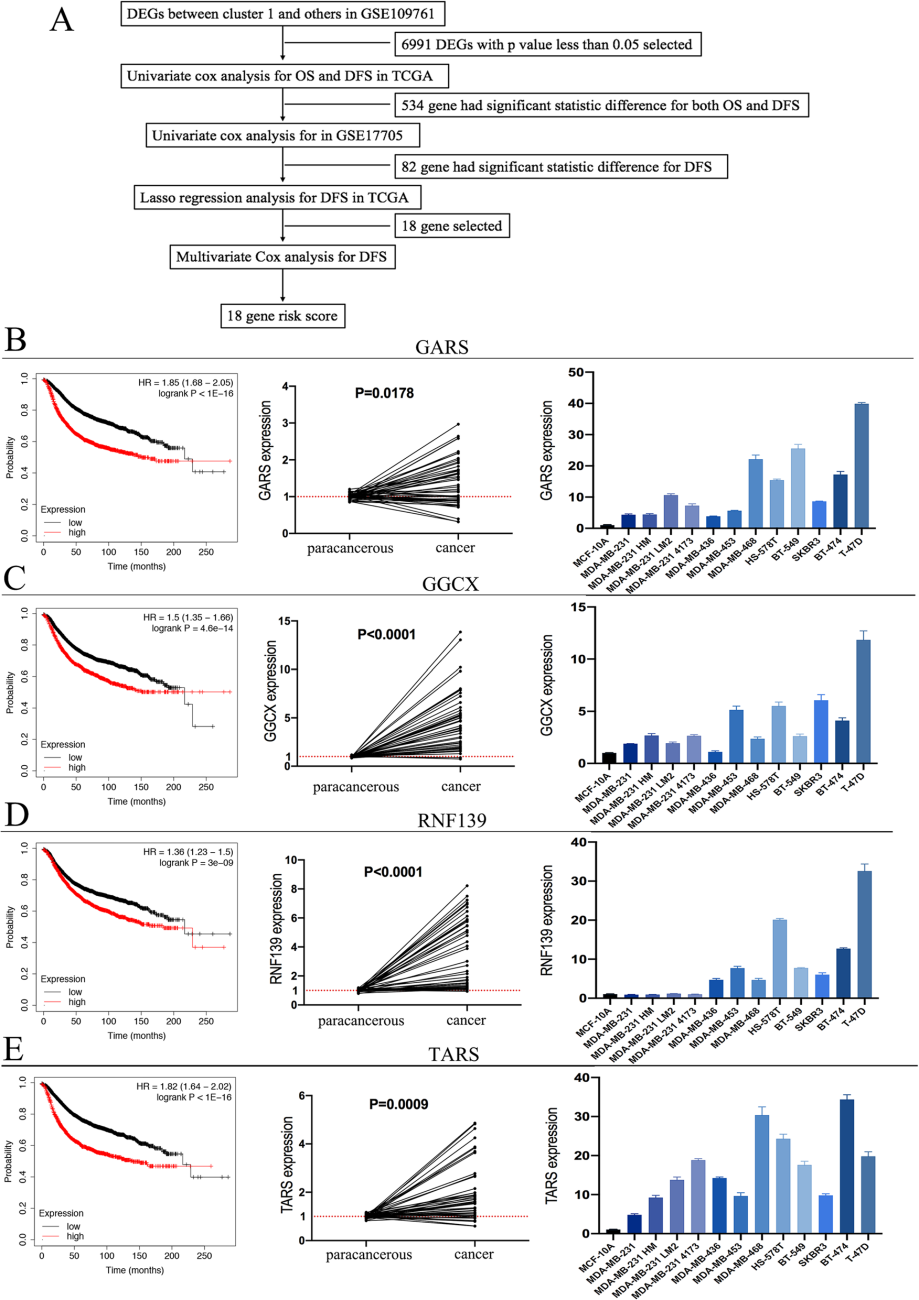
Constitution of risk score and its verification. **A** Flow chart of the 18-risk score constituting gene selection. Kaplan–Meier (K–M) curves show the recurrence-free survival of patients with high and low expression of GARS (**B**), GGCX (**C**), RNF139 (**D**), and TARS (**E**) (picture on the left). The gene expression of GARS (**B**), GGCX (**C**), RNF139 (**D**), and TARS (**E**) in 16 pairs of matched breast carcinomas and paraneoplastic tissues (picture on the medium). Gene expression of GARS (**B**), GGCX (**C**), RNF139 (**D**), and TARS (**E**) in breast cancer cell lines (picture on the right)

Considering the convenient application of special CTC clusters in solid breast cancer, we wanted to develop a user-friendly gene set based on the differentially expressed genes between Cluster 1 and others of GSE109761. To identify special gene expression profiles of Cluster 1, we used the "limma" package and identified 6991 differentially expressed genes by comparing Cluster 1 and others. We then applied the 6991 selected genes in bulk RNA-Seq data of breast cancer from TCGA. After univariate Cox analysis, 534 genes with significant differences in OS and DFS were selected. To obtain more stable results, we used other RNA-Seq data from GSE17705 to further validate the relationship between genes and survival, and we found 82 genes from the 534 genes were significantly different for DFS. Lasso regression analysis was applied using the 82 genes in the TCGA cohort, and 18 genes were obtained with a high association with patients DFS (Fig. 2A). Among these 18 genes, four oncogenes, GARS, GGCX, RNF139, and TARS, were not previously reported to be related to breast cancer. Large gene chip data and clinical conjoint analysis show that the high expression of these four genes correlated with short recurrence-free survival in breast cancer (picture on the left of Fig. 2B (GARS), C (GGCX), D (RNF139), and E (TARS)). We analyzed gene expression in 16 pairs of matched carcinomas and paraneoplastic tissues of our hospital and found that these four genes were expressed at significantly higher level in carcinoma tissues than in paraneoplastic tissues (picture on the medium of Fig. 2B (GARS), C (GGCX), D (RNF139), and E (TARS)). The gene expression result in breast cancer cell lines showed that these four genes were expressed at higher levels in breast cancer cells than in normal mammary epithelial cells (picture on the right of Fig. 2B (GARS), C (GGCX), D (RNF139), and E (TARS)).

### GARS is an oncogene for breast cancer

**Fig. 3:**
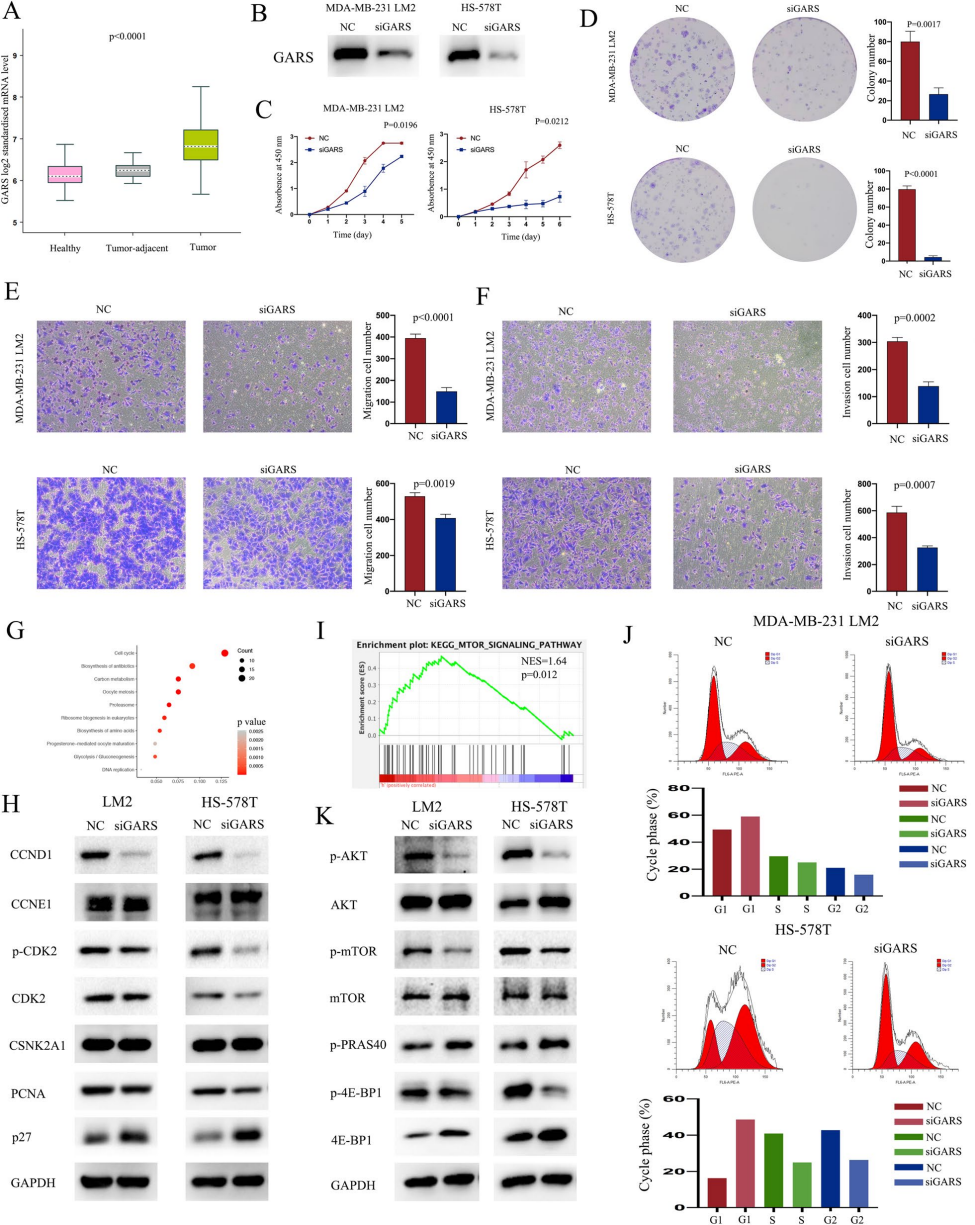
GARS is an oncogene for breast cancer. **A** The expression of GARS in breast cancer, tumor-adjacent tissues, and normal tissues from the GTEx and TCGA datasets. **B** Validation of GARS knockdown efficacy in breast cancer cell lines by Western blot. **C** Cell proliferation capacity was examined in control and GARS knockdown cell lines by CCK-8 assay. **D** A colony formation assay was carried out to evaluate the proliferation abilities of breast cancer cells in control and GARS knockdown conditions. Cell migration assay (**E**) and invasion assay (**F**) for HS-578T cells and MDA-MB-231 LM2 in control and GARS knockdown conditions. **G** GSEA found that mTOR signaling pathway genes were enriched in the high GARS expression group. **H** Western blot analysis of AKT-mTOR signaling pathway proteins in control or GARS knockdown cells. **I** KEGG analysis of the most positively related genes of GARS. **J** Cell cycle analysis was performed with flow cytometry in cells (control or GARS knockdown cells) after release at the same time. **K** Western blot analysis of cell cycle-related proteins in control or GARS knockdown cells

We further chose GARS to explore its function in breast cancer. The data obtained from TCGA and GTEx demonstrated that GARS was overexpressed in breast cancer tissues compared with tumor-adjacent tissues and healthy tissues, which is consistent with our hospital results (Fig. 3A, P < 0.0001). From the results of GARS RNA expression in breast cancer cell lines, we chose HS-578T and MDA-MB-231 LM2 cells to knock down GARS expression by siRNA. The knockdown efficiency of GARS was analyzed by Western blot, and siRNA significantly decreased GARS expression in cells (Fig. 3B). A CCK-8 assay was performed to analyze the potential effect of GARS on cell proliferation. The results reflect that knockdown of GARS in breast cancer cells significantly inhibited cell proliferation (Fig. 3C). The colony formation assay showed that knockdown of GARS weakened the colony formation ability of MDA-MB-231 LM2 cells and HS-578T cells (Fig. 3D). In the migration assays, the number of cells that crossed the membrane was significantly decreased in GSRA knockdown cells (Fig. 3E). These results were similar in the invasion assays, in which the number of cells that crossed the Matrigel was significantly decreased in GSRA knockdown cells in both cell lines (Fig. 3F). These results indicated that GARS promotes the proliferation and invasion capacity of breast cancer.

### GARS controls the mTOR signaling pathway to promote breast cancer progression

To explore the GARS-related pathway that impacts its ability to promote tumor progression, we used GSEA to identify the most enriched pathways in the high GARS group in the TCGA dataset. The mTOR signaling pathway genes showed significant enrichment in the high GARS expression group (Fig. 3G). Thus, we used Western blotting to verify the classical proteins in the mTOR signaling pathway. AKT and mTOR phosphorylation levels were decreased in GARS knockdown cells compared with control cells. EIF4EBP1 (4E-BP1) is an important downstream target of mTOR and controls the mRNA translation of many tumor progression-related genes. The phosphorylation level of 4E-BP1 was decreased in GARS knockdown cells, while the level of total 4E-BP1 in these cells was increased, which means that the mRNA translation process was inhibited in GARS knockdown cells (Fig. 3H). These findings suggested that GARS promotes breast cancer progression by activating the PI3K/AKT/mTOR pathway.

### GARS accelerated the cell cycle of breast cancer

KEGG analysis using the most positively related genes of GARS from the TCGA dataset revealed many tumor-related pathways, including the cell cycle (Fig. 3I). As GARS regulates cell proliferation, we started to explore the potential underlying mechanism. We first examined cell cycle patterns in control and GARS knockdown cells. As shown in Fig. 3J, GARS knockdown cells had a higher proportion of G0/G1 phase cells and a lower proportion of S and G2 phase cells after release for some time. These results revealed that GARS knockdown prevents cells from entering the S phase from the G0/G1 phase. Cell cycle-regulating proteins were measured by Western blot to examine whether GARS knockdown truly alters the expression of cell cycle checkpoint proteins. The results revealed that knockdown of GARS significantly downregulated the expression of CCND1, CDK2, phosphorylated CDK2, and PCNA; however, knockdown of GARS upregulated the expression of the cell cycle-inhibiting protein P27 (Fig. 3K). These results suggest that GARS may regulate the cell cycle by upregulating CDK2 and CyclinD1 expression in breast cancer.

### The risk score construction

**Fig. 4:**
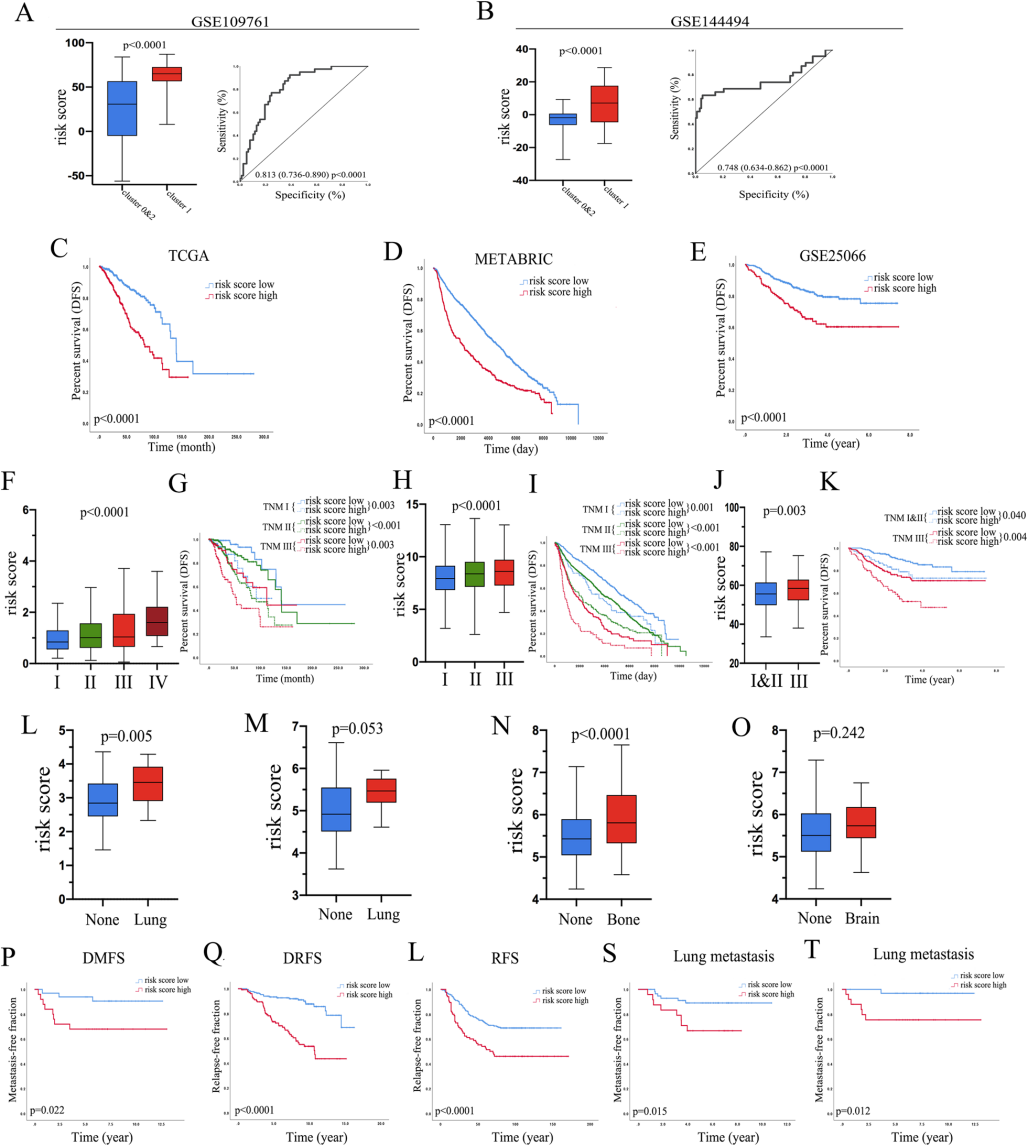
The risk score correlated with aggressive clinicopathology types in breast cancer. The mean value of the risk score in Cluster 1 or others in GSE109761 (**A**; left picture) and GSE144494 (**B**; left picture). Receiver operating characteristic curve (ROC) analysis of the risk score for detecting Cluster 1 patients in GSE109761 (**A**; right picture) and GSE144494 (**B**; right picture). Kaplan–Meier curves for DFS according to the risk score classification in TCGA (**C**), METABRIC (**D**), and GSE25066 (**E**). The mean value of the risk score in different TNM stages in the TCGA (**F**), METABRIC (**H**), and GSE25066 (**J**) cohorts. Reclassification of survival curves according to the risk score classification within each TNM stage in the TCGA (**G**), METABRIC (**I**), and GSE25066 (**K**) cohorts. Boxplots show the risk score of primary tumors and metastases of patients who later developed metastasis to the lung (**L**: GSE2603; M: GSE5327), bone (**N**: GSE2034), or brain (**O**: GSE2034). The Kaplan–Meier survival plots depict metastasis-free survival for distant metastasis (**P**: GSE5327), distant relapse (**Q**: GSE17707), total relapse (**L**: GSE2034), and lung metastasis (**S**: GSE2603); (**T**: GSE5327) based on the premetastatic primary tumor risk score classification

Based on gene expression and regression coefficient values from multivariate Cox analysis of the 18 genes, we established a simple risk score = (COPS5 * 1.694701 + CPT1A * 1.608622 + GARS * 1.411987 + GGCX * 0.903292 + HCCS * 0.929408 + HMGB3 * 1.229127 − KRT15 * 0.492186 − N4BP2L1 * 0.320413 − PRKCB * 1.003909 + RNF139 * 0.957138 − RPS18 * 6.077616 + SCARB2 * 3.14392 − SERPINA1 * 1.783167 + SHMT2 * 1.226157 + TARS * 2.705961 + TNFRSF14 * 0.634075 − TOR1B * 0.247428 + TXN * 2.918599).We applied the risk score in circulating tumor cell cohorts, and the epithelial cell cluster (Cluster 1 in GSE109761 (Fig. 4A (left picture)); Cluster 1 in GSE144494 (Fig. 4B (left picture))) had a significantly higher risk score than the others. The area under the curve (AUC) of the receiver operating characteristic (ROC) curve that shows the relationship of the risk score and epithelial cell cluster was 0.813 (p < 0.0001) in GSE109761 (Fig. 4A (right picture)) and 0.750 (p < 0.0001) in GSE144494 (Fig. 4B (right picture)). These results mean that the risk score classification had high consistency with the special epithelial cell cluster (Cluster 1) in CTC cohorts.

### The risk score related to high metastasis and poor survival in breast cancer

We used the risk score in different breast cancer cohorts, and the DFS was significantly lower in the high-risk score group than in the low-risk score group, as shown in Kaplan–Meier (K-M) curves (TCGA (Fig. 4C); METABRIC (Fig. 4D); GSE25066 (Fig. 4E)). Univariate and multivariate Cox analyses demonstrated that the risk score was an independent prognostic factor for survival in breast cancer (Table 1). We applied the risk score classification to different cohorts and found that the risk score was associated with some clinicopathological features, such as the TNM stage. Box diagrams show that the risk score was higher in high TNM stages (TCGA (Fig. 4F); METABRIC (Fig. 4H); GSE25066 (Fig. 4J)). We know that the TNM stage is an important prognostic signature for breast cancer. To investigate whether the risk score classification could reclassify the TNM stage, we applied the risk score classification in each TNM stage. The risk score classification could reclassify patients in TNM stage 1 into two significantly different DFS groups, as well as in TNM stages 2 and 3 (TCGA cohort (Fig. 4G), METABRIC cohort (Fig. 4I) and GSE25066 cohort (Fig. 4K)).

**Table 1:** Univariate and Multivariate Cox Analysis of variables with DFS

Variables	Univariate analysis		Multivariate analysis	
	HR (95%CI)	p	HR (95%CI)	p
TCGA				
Age (> 50 vs ≤ 50)	1.293 (0.885–1.889)	0.184		
T		< 0.0001		0.007
T1	1 (reference)		1 (reference)	
T2	1.525 (0.967–2.405)		1.138 (0.710–1.825)	
T3	2.521 (1.436–4.424)		1.928 (1.062–3.500)	
T4	9.417 (4.725–18.767)		3.178 (1.441–7.006)	
N		< 0.0001		0.003
N0	1 (reference)		1 (reference)	
N1	1.416 (0.919–2.184)		1.152 (0.738–1.797)	
N2	2.821 (1.733–4.594)		1.748 (1.024–2.986)	
N3	4.246 (2.446–7.370)		2.976 (1.611–5.498)	
Molecular subtypes		0.009		0.088
Luminal A&B	1 (reference)		1 (reference)	
HER2 positive	2.902 (1.346–6.258)		2.041 (0.926–4.498)	
Triple negative	1.535 (0.949–2.485)		1.469 (0.884–2.442)	
Risk score (high vs low)	2.833 (1.994–4.025)	< 0.0001	2.506 (1.723–3.644)	< 0.0001
METABRIC				
Age (> 50 vs ≤ 50)	1.403 (1.204–1.635)	< 0.0001	1.326 (1.134–1.551)	< 0.0001
T		< 0.0001		< 0.0001
T1	1 (reference)		1 (reference)	
T2	1.559 (1.381–1.759)		1.371 (1.211–1.552)	
T3	2.316 (1.804–2.974)		1.784 (1.381–2.305)	
N		< 0.0001		< 0.0001
N0	1 (reference)		1 (reference)	
N1	1.321 (1.162–1.501)		1.245 (1.093–1.418)	
N2	2.162 (1.769–2.641)		1.887 (1.540–2.314)	
N3	4.144 (3.300–5.203)		3.411 (2.699–4.311)	
Molecular subtypes		0.099		0.857
Luminal A&B	1 (reference)		1 (reference)	
HER2 positive	1.244 (0.986–1.570)		1.011 (0.793–1.288)	
Triple negative	0.925 (0.783–1.094)		0.955 (0.805–1.132)	
Risk score (high vs low)	1.413 (1.255–1.591)	< 0.0001	1.294 (1.145–1.463)	< 0.0001
GSE25066				
Age (> 50 vs ≤ 50)	1.072 (0.728–1.579)	0.723		
T		< 0.0001		0.049
T1	1 (reference)		1 (reference)	
T2	1.520 (0.470–4.914)		1.472 (0.453–4.776)	
T3	2.200 (0.673–7.188)		1.788 (0.544–5.877)	
T4	4.370 (1.326–14.404)		2.885 (0.857–9.714)	
N		< 0.0001		0.002
N0	1 (reference)		1 (reference)	
N1	2.670 (1.504–4.741)		2.271 (1.275–4.048)	
N2	5.088 (2.650–9.768)		3.689 (1.881–7.236)	
N3	4.162 (1.944–8.911)		2.509 (1.142–5.515)	
Molecular subtypes		< 0.0001		< 0.0001
Luminal A&B	1 (reference)		1 (reference)	
HER2 positive	3.734 (0.513–27.197)		3.854 (0.510–29.123)	
Triple negative	3.519 (2.365–5.236)		3.157 (2.108–4.729)	
Risk score (high vs low)	2.159 (1.452–3.209)	< 0.0001	1.948 (1.304–2.910)	0.001

The variables with p < 0.1 in univariate Cox Regression Analysis were selected for the further multivariate Cox Regression Analysis

*DFS* disease free survival, *HR* hazard ratio, *CI* confidence interval, *vs* versus

We examined the risk scores of primary and lung metastasis tumors in the GSE2603 and GSE5327 cohorts. The risk score was significantly elevated in lung metastasis tumors compared with the primary tumors, as shown in Fig. 4L (GSE2603) and M (GSE5327). In the GSE2034 cohort, patients with bone metastasis had a higher risk score than patients with primary breast cancer (Fig. 4N), and patients with brain metastasis also had a higher risk score than patients with primary breast cancer, although the difference was not statistically significant (Fig. 4O). The K-M curves show that the high-risk score was associated with high distant metastasis risk (Fig. 4P; GSE5327), distance relapse (Fig. 4Q; GSE17707), and total relapse (Fig. 4L; GSE2034) events. Site-specific metastasis-free survival demonstrated that the risk score was significantly associated with lung metastasis (Fig. 4S (GSE2603) and T (GSE5327)).

### Mutation variations between low- and high-risk score groups

**Fig. 5:**
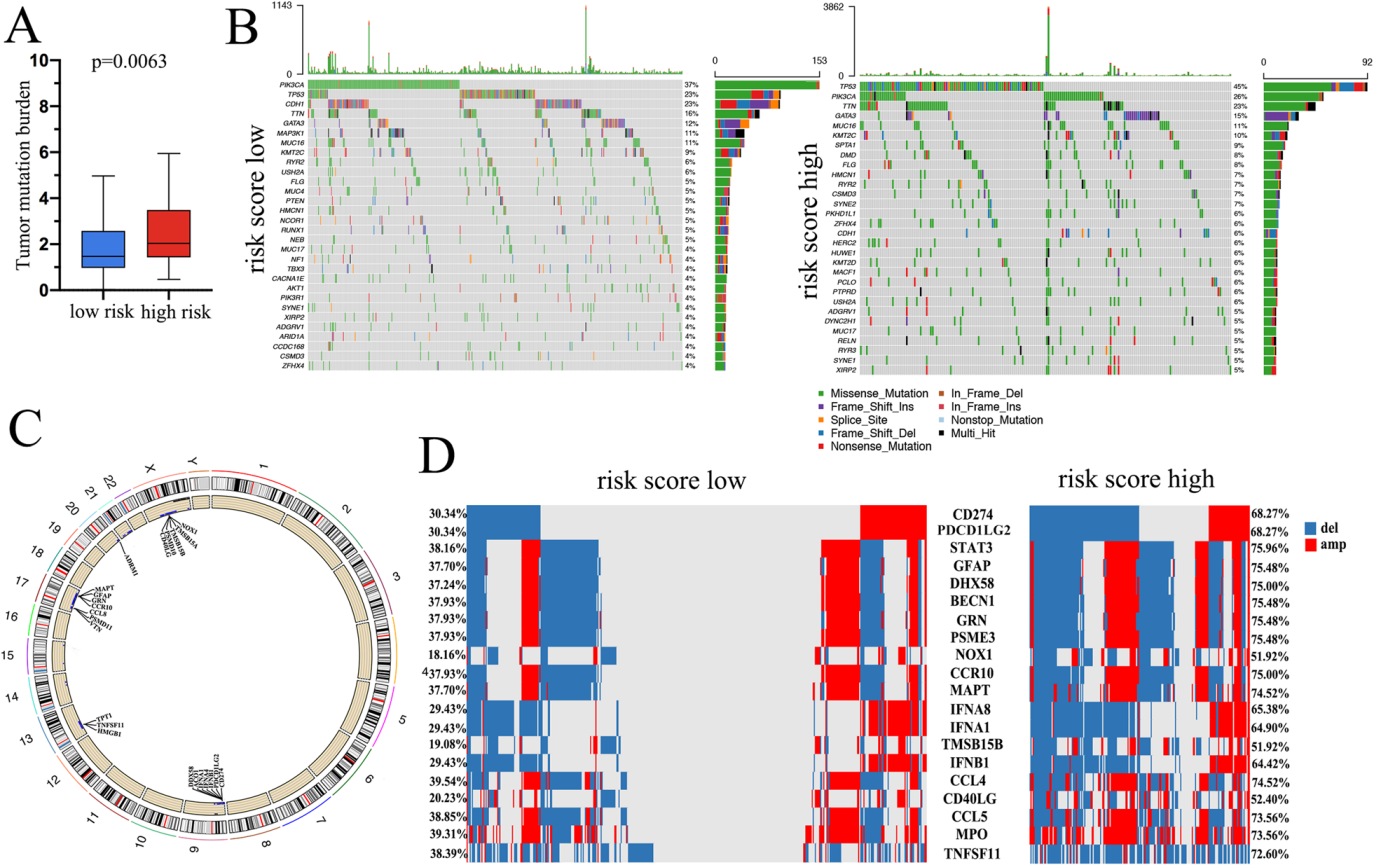
Risk score associated with tumor mutation. **A** The tumor mutation burden (TMB) in the low- and high-risk score groups in the TCGA cohort. **B** The waterfall plot shows the top 30 genes with the highest mutation rates in the low- and high-risk score groups in TCGA. **C** Chromosome sites of some immune-related genes with high copy number alteration (CNA) rates. **D** Immune-related genes with significantly high CNA rates in the low- and high-risk score groups

We analyzed the total tumor mutation burden (TMB) of the risk score classification and found that the high-risk score group had higher TMB than the low-risk score group (Fig. 5A). We then explored the most frequently mutated genes in the low- and high-risk score groups separately. The waterfall plot shows the top 30 genes with high mutation rates in each risk group, and the most mutated genes were somewhat different. The top mutated gene in the low-risk score group was PIK3CA, which was mutated in 37% of patients in this group, while the mutation rate was 26% in the high-risk score group (Fig. 5B). The top mutated gene in the high-risk score group was TP53, which was mutated in 45% of patients in this group, while the rate was 23% in the low-risk score group (Fig. 5B). By using the GISTIC, we analyzed copy number alterations between the high- and low-risk score groups. The high-risk score group sustained a significantly higher CNA rate for most genes than the low-risk score group (results not shown). We found that some immune-related genes had significantly high CNA rates in the high-risk score group, and these immune gene-related CNA changes occurred mostly on chromosomes 9, 17 and X (Fig. 5C, D).

### The relationship of risk score and immune features

**Fig. 6:**
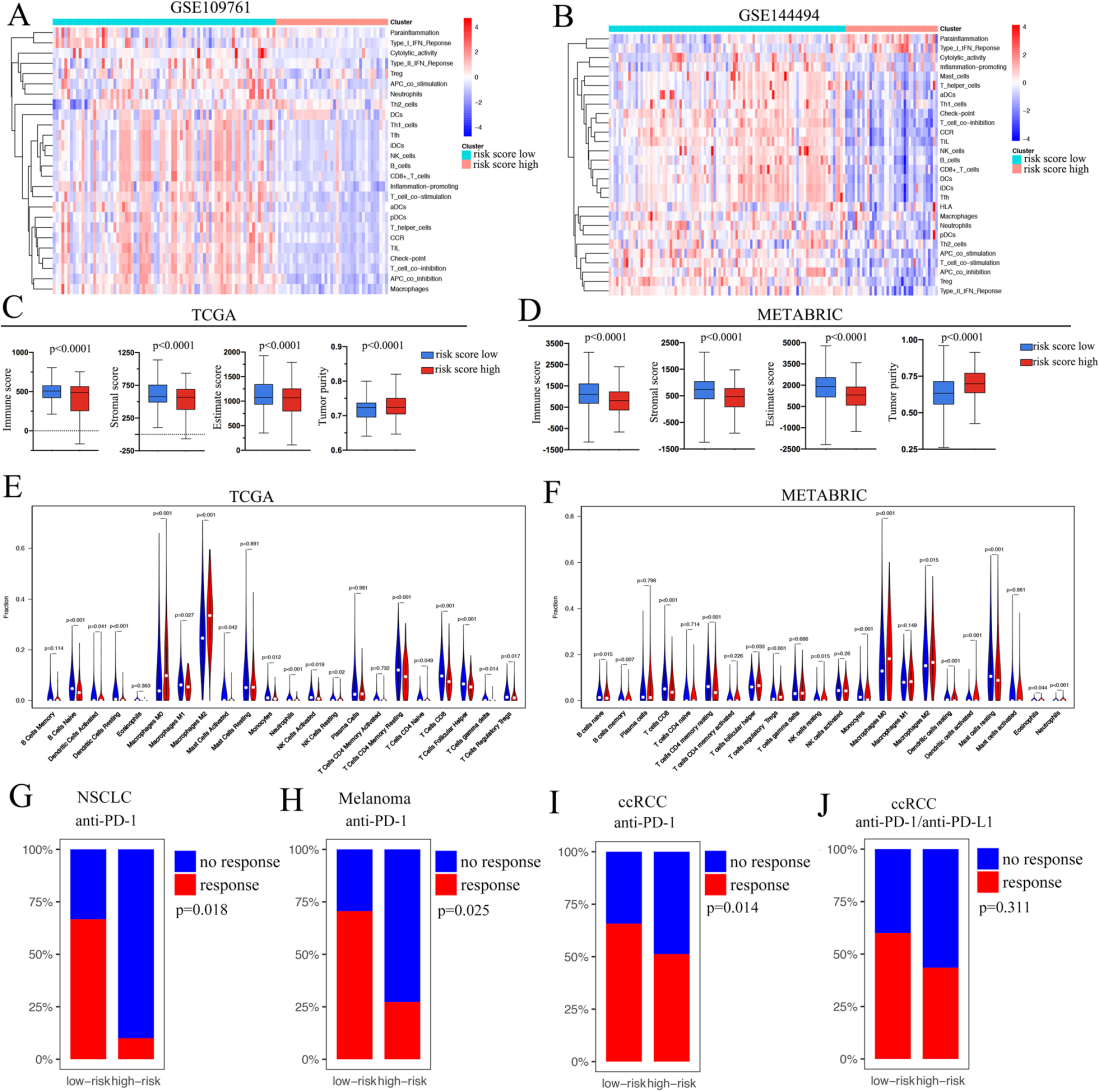
Risk score related to a low immune infiltration environment in breast cancer. **A** Unsupervised clustering of ssGSEA scores of immune signatures in the single-CTC cohort **A** GSE109761 and **B** GSE144494 according to the risk score classification. The box diagram shows the immune score, stromal score, ESTIMATE score, and tumor purity in the low- and high-risk score groups in the TCGA (**C**) and METABRIC (**D**) cohorts. Comparison of the fraction of twenty-two types of immune cells between the low- and high-risk score groups in the TCGA (**E**) and METABRIA (**F**) cohorts. **G**–**J** The PD-1/PD-L1 therapy response rate in the low- and high-risk score groups verified in four separate cohorts

To explore the relationship between risk score and immune features, we applied ssGSEA in single-CTC sets GSE109761 and GSE144494. The high-risk score group showed significantly low enrichment in most immune features, such as NK cells, B cells, and neutrophils, etc. (Fig. 6A, B). These results indicate that the high-risk score group may belong to the immune deficient state. We then analyzed the immune states of risk score classification in solid breast cancer samples. In both the TCGA and METABRIC cohorts, we found that the immune score, stromal score, and estimate score were significantly lower in the high-risk score group than in the low-risk score group. However, the tumor purity was higher in the high-risk score group (Fig. 6C, D). The fractions of twenty-two types of immune cells were analyzed in the TCGA and METABRIA cohorts separately, and infiltrated T cells, B cells, neutrophils, and NK cells were significantly lower in the high-risk score group, while the macrophage types 0 and 2 were statistically higher in the high-risk score group (Fig. 6E, F).

As we found that the risk score was negatively correlated with some immune features, including immune checkpoints, we sought to determine whether the risk score could predict the immunotherapy response. We applied our risk score in four independent datasets in which the patients received immunotherapy. As expected, the high-risk score group contained fewer therapy response patients in all four sets (Fig. 6G: Cho et al. [19]; Fig. 6H: Hugo et al. [20]; Fig. 6I: Braun et al. [21]; Fig. 6J: Diana Miao et al. [22]), which means that the high-risk score patients may not be sensitive to immunotherapy.

### Pathway analysis of the risk score

**Fig. 7:**
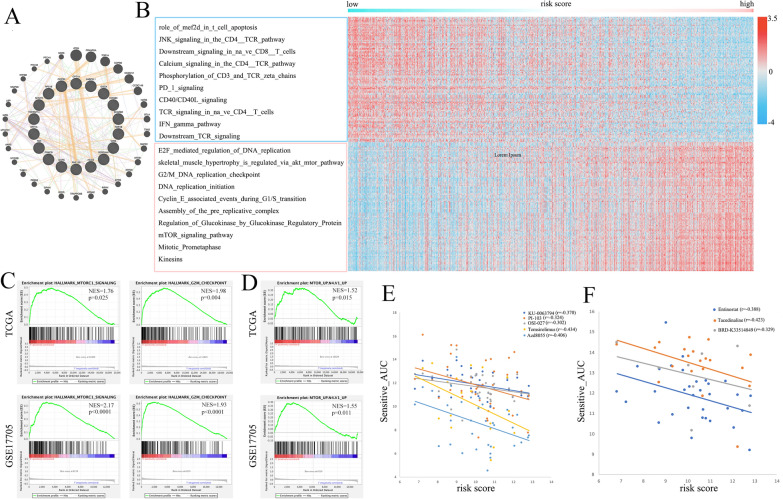
Bioinformatics and drug sensitivity analysis of the risk score. **A** The 18 genes that make up the risk score and their most correlated genes. **B** The Pearson correlation of ssGSEA scores of pathways from NCI-PID, BioCarta, and Reactome datasets with our risk score. **C** GSEA of HALL-MARK pathway sets in the TCGA and GSE17705 cohorts. **D** GSEA of oncogene signature set in the TCGA and GSE17705 cohorts. **E** The scatter plot shows the drug-sensitive correlation of the risk score and PI3K/AKT/mTOR inhibitors in the CTRP dataset. **F** The scatter plot shows the drug-sensitive correlation of the risk score and CDK inhibitors in the CTRP dataset

The 18 genes that make up the risk score and their most correlated genes are shown in Fig. 7A. We compared the ssGSEA scores of 1387 constituent pathways from three pathway databases, NCI-PID, BioCarta, and Reactome, with our risk score. The risk score was positively correlated with mTOR signaling, CDK regulation of DNA replication signaling, mechanism of protein import into the nucleus signaling, etc. In contrast, the risk score was negatively related to immune-related pathways such as JNK signaling in the CD4_TCR pathway, phosphorylation of CD3 and TCR zeta chains, Downstream TCR signaling, etc. (Fig. 7B). The hallmark gene signature set of GSEA in TCGA and GSE17705 shows that the genes in the high-risk score group were enriched in the MTORC1 SIGNALING and G2M CHECKPOINT SIGNALING pathways, which is consistent with the ssGSEA results (Fig. 7C). The oncogene signature set of GSEA in TCGA and GSE17705 also showed that the mTOR signaling pathway was enriched in the high-risk score group (Fig. 7D).

### Risk score and drug-sensitive analysis

As the pathway analysis revealed that the high-risk group was active in the PI3K-AKT-mTOR and CDK pathways, we used the gene expression and drug sensitive_AUC data from the CTRP dataset to validate the effect of those two pathway inhibitors on the risk score. The scatter plot shows that the risk score was negatively related to PI3K (Fig. 7E) and CDK pathway (Fig. 7F) inhibitors, which means that the higher the risk score was, the more sensitive these drugs were.

## Discussion

In this study, we used scRNA-seq of CTCs to identify a cluster of epithelial cells that had more aggressive characteristics than other CTCs in breast cancer. Applying the differentially expressed genes selected from the single-CTCs clusters in bulk sequence data of solid breast cancers, we identified 18 genes that were closely related to breast cancer patient prognosis. The risk score established by the 18 genes had a strong association with DFS and OS in breast cancer that was verified by a series of datasets. We explored the relationship of the risk score and tumor immune infiltration features by many methods and found that the high-risk score related to a defective immune infiltration environment and immune checkpoint therapy response rate were lower in the high-risk score patients. The GSEA shows that the genes in the high-risk score group were enriched in the mTOR, and G2M CHECKPOINT SIGNALING pathways, and the subsequent drug-sensitive analysis shows that the high-risk score patients may be more sensitive to AKT-mTOR and CDK pathway drugs.

Extraction and analysis of liquid biomarkers such as CTCs is a noninvasive method that can screen tumors and determine the prognosis or drug sensitivity of cancer patients. Recently, scientists have tried many techniques to isolate single or cluster CTCs from the blood of patients, and with the improvement of deep-seq techniques, single-CTC RNA could be qualitatively compatible with single-cell RNA sequencing tests [23–26]. The development of these techniques made use and thorough analysis of CTCs possible. Tumor cells leave the primary focus in two main ways and enter the blood circulation to become CTCs through epithelial-mesenchymal transition (EMT) of cells or directly enter the blood through the gap of endothelial cells of the neovasculature [27, 28]. CTCs play an important role in the metastasis of cancers, but they also face some threats, such as immune cell recognition and killing or blood flow shear force, that may eliminate them [29]. The molecules expressed in CTCs could protect them from recognition by immune cells. Beccelli et al. and his colleagues found that CD47 and other protein expression on the CTC surface can reduce immune cell killing of CTCs [30]. Low expression of human leukocyte antigen class I (HLA-I) protein in CTCs has been found in many cancers and is correlated with aggressive malignancy of tumors, and in some types of cancer, it is also related to the low response rate to treatment [31, 32]. CTC molecular and mechanistic dissection can help us explore the heterogeneity of tumors and promote the clinical use of tumor CTCs. Here, the gene expression results of CTCs from breast cancer and a more detailed classification were analyzed in our study. We used different expression marker genes in CTCs to identify a cluster of cells that highly expressed cancer-associated cell-surface markers and expressed low level of HLA-I class proteins and other proteins, such as CD47. As expected, the cluster of cells showed more aggressive characteristics both in pathway analysis and Kaplan–Meier curve survival analysis. The subsequent analysis based on the differentially expression genes of the identified cluster CTCs and others could be more reliable.

By comprehensive analysis of gene expression in CTCs and bulk solid breast cancer datasets, we determined 18 genes that had a strong survival relationship with breast cancer. Some of these 18 genes were previously reported to be related to breast cancer prognosis. COPS5, also known as CSN5, is a component part of the COP9 signalosome, but its amplification is required for primary human breast epithelial cell malignant transformation [33]. SERPINA1 is a suppressive gene in breast cancer and is the target gene directly regulated by PIWI-interacting small noncoding RNAs (piR-36026) and the response to molecular therapy [34]. SHMT2 catalyzes the first step of one-carbon metabolism, and the high expression of SHMT2 was significantly correlated with poor survival in breast cancer [35, 36]. Among these 18 genes, some were reported to influence the tumor immune environment. COPS5 was found to stabilize PD-L1 in breast cancer through deubiquitination of the PD-L1 molecule [37]. TNFRSF14 (HVEM) has been observed to have a prognostic impact in breast cancer depending on the level of tumor-infiltrating lymphocytes (TILs), and the worst outcome occurs in patients with high TNFRSF14 expression and low TIL tumors [38]. Interestingly, some of the genes included in the 18 genes were not reported to be major markers in breast cancer, such as GARS, GGCX, RNF139, and TARS, etc. which can help us to find new prognostic markers for further analysis.

In this study, we selected GARS for further analysis of its function in breast cancer. GARS is a glycyl-tRNA synthetase and is related to protein synthesis and neddylation [39]. The function of GARS in cancer has been reported in only a few tumors. Chen et al. used iTRAQ proteomics technology in samples from urothelial carcinoma and chronic kidney disease patients and found that GARS was more highly expressed in urothelial carcinoma and could be used as a diagnostic marker for urothelial carcinoma [40]. Rahane et al. reported that the mutation of GARS may play an oncogenic driver role in adrenocortical carcinoma [41]. We found that GARS act as an oncogene in breast cancer. Inhibition of GARS decreased the cell growth, colony formation, migration, and invasion of breast cancer cells. Bioinformatics analysis and Western blot analysis helped us find that GARS may influence the malignant progression of breast cancer through the AKT/mTOR pathway. Knockdown of GARS also blocks the cell cycle in breast cancer. The cell cycle inhibitor kinase p27 was significantly higher in GARS knockdown cells. As GARS related to neddylation and p27 could be degraded by ubiquitin progression, we speculate that GARS may improve p27 degradation, which accelerates the cell cycle in breast cancer [42]. These experiments verified the important role of GARS in breast cancer and the accuracy of the breast cancer-related markers that we found by using multiomics.

We used 18 genes to build a risk score for breast cancer. Intratumoral heterogeneity in breast cancer has been well documented, and subclones consist of distinct genotypes that have distinct behaviors. Detailed classification of tumors at the clinical and genetic levels is helpful for individual treatment making. In recent years, with the development of in-depth sequencing technology and the removal of restrictions to use this technology for researchers, many attempts have been made to identify special gene cohorts of tumors to better predict the outcomes of patients. MammaPrint relies on 70 gene expression data points from microarray-based measurements and is used for predicting the prognosis of early-stage breast cancer patients [43]. Oncotype DX, which includes 21 genes, was designed to predict the benefit of chemotherapy in early-stage invasive breast cancer patients with the ER-positive/HER2-negative type [44]. Our 18-genes score can be applied in all types and stages of breast cancer patients, and a high score indicates a high metastasis tendency and poor survival rate. The TNM staging system, due to its practice and simplicity, is the most widely used cancer staging system [45]. The TNM stage can classify patients with breast cancer into roughly four prognostic groups. We applied our 18-genes risk score in each TNM stage group and found that the risk score can better reclassify the same group of patients into a more accurate risk level, which means our risk score can add prognostic and predictive information to classical parameters for breast cancer patients.

We compared the mutation variations between the low- and high-risk score groups and found that higher TMB and TP53 mutations occurred in the high-risk score group. TP53 mutation correlated with high epigenomic instability and poor prognosis in breast cancer, which can partly explain why the high-risk score group patients had aggressive malignancy behavior. The copy number variation between the high- and low-risk score groups also showed a significant difference. Some immune-related genes had higher CNA rates in the high-risk score group, such as CD274 (PD-L1) and PDCD1LG2 (PD-L2). In our study, the high-risk score group had high deletion of PD-L1/PD-L2 genes. A previous study revealed that amplification of PD-L1 and PD-L2 are important biomarkers for immunotherapy, Gupta et al. found that the amplification of PD-L1/PD-L2 may play a potential mechanism of resistance to chemotherapy in breast cancer [46]. These results imply that the high-risk score group patients may benefit less from immune checkpoint treatment and benefit more from chemotherapy, which is consistent with our drug-sensitive analysis.

Immune checkpoint inhibitor (ICI) therapies have been successfully applied in many tumors and opened a promising new way for cancer therapy [47]. Some ICI therapies have also been attempted in breast cancer, such as atezolizumab, which was approved for use in breast cancer by combination with nab-paclitaxel [48]. Many attempts have been made to identify patients who could benefit most from ICI treatment. The tumor immune microenvironment is critical to the ICI treatment response, and researchers have analyzed it in pan-cancer of TCGA dataset [49]. Tumors with an immune-excluded and immune-desert phenotype rarely respond to anti-PD-1/PD-L1 therapy because of active T-cell exclusion or lack in the tumor parenchyma or stroma [50, 51]. The immune-inflamed type of tumor has abundant adaptive and innate immune cell infiltration, and it predicts a significantly better ICI therapy response and good survival prognosis in tumors [51, 52]. In our study, the risk score shows a significant correlation with the tumor immune micro-environment. The data show that the risk score was negatively correlated with the infiltration of most immune cell types, including TILs, NK cells, and CD8+ T cells, etc. These results appeared not only in breast cancer but also in other solid tumors, revealing its universal application in tumors. We analyzed the response rate of ICI treatments according to our risk score classification and found that the high-risk score group had a significantly lower response rate than the low-risk score group in many kinds of tumors. All these findings indicate that the high-risk score group patients tended to have immune-excluded or immune-desert phenotypes, so ICI treatment may not be useful for them.

In the comprehensive pathway analysis through KEGG, GSEA, and ssGSEA, the PI3K/AKT/mTOR pathway and cell cycle regulatory-related pathways were significantly active in the high-risk score group. The PI3K/Akt/mTOR signaling pathway has been found to be hyperactive in almost all tumors, including breast cancer [53]. This pathway is involved in many cellular activities, such as cell growth, proliferation, survival, metabolism, and immune response regulation [54, 55]. The dysregulation of this pathway leads to uncontrolled cell proliferation, genomic instability, and metabolic reprogramming that promote the malignant development of tumors [56]. Cell cycle regulatory related pathways tightly regulate each cell cycle phase in normal cells [57]. Once dysregulated, these pathways may induce breast epithelial cells to transform to the active state, leading to oncogenic changes [58]. The abnormal activation of these pathways partly explained the aggressive behavior of the high-risk score group patients. Therefore, blocking PI3K/AKT/mTOR and the cell cycle pathway may be helpful for risk score-high breast cancer patients, and the subsequent drug-sensitive analysis targeted to these pathways confirmed this.

In summary, we identified an aggressive cluster of single CTCs of breast cancer based on its distinct gene expression pattern. By using the differentially expressed genes selected from the single-CTCs clusters, we constructed an 18-gene risk score in bulk solid breast cancer datasets. The risk score classified patients into distinct metastasis and survival prognosis groups, and their immune cell infiltration status was also different. Immune checkpoint inhibitors may not be sensitive to risk score-high patients, while drugs that target the abnormally activated pathways in this group, such as the PI3K/AKT/mTOR pathway and cell cycle regulated pathways, may be helpful for those patients. Our risk score contains fewer genes and can easily be applied in the clinic. We hope in the future, that there will be large-scale prospective studies to validate our results.

## Supplementary Information

**Additional file 1: Table S1.** Primary antibodies used in our study.

## Data Availability

The datasets used and analyzed during the current study are available from the Gene Expression Omnibus (GEO) (https://www.ncbi.nlm.nih.gov/geo/), The Cancer Genome Atlas (TCGA) (https://portal.gdc.cancer.gov/) and METABRIC cohort (https://www.cbioportal.org/).
